# A Rapid Point-of-Care Test for the Serodiagnosis of Hepatitis Delta Virus Infection

**DOI:** 10.3390/v13122371

**Published:** 2021-11-26

**Authors:** Florian A. Lempp, Imme Roggenbach, Shirin Nkongolo, Volkan Sakin, Franziska Schlund, Paul Schnitzler, Heiner Wedemeyer, Frédéric Le Gal, Emmanuel Gordien, Cihan Yurdaydin, Stephan Urban

**Affiliations:** 1 Department of Infectious Diseases, Molecular Virology, University Hospital Heidelberg, 69120 Heidelberg, Germany; f.lempp@gmx.net (F.A.L.); imme.roggenbach@gmx.de (I.R.); shirin.nkongolo@med.uni-heidelberg.de (S.N.); vsakin@gmail.com (V.S.); franziska.schlund@med.uni-heidelberg.de (F.S.); 2 German Centre for Infection Research (DZIF), 69120 Heidelberg, Germany; 3 Toronto Centre for Liver Disease, University Health Network, Toronto, ON M5G 1L7, Canada; 4 Department of Infectious Diseases, Virology, University Hospital Heidelberg, 69120 Heidelberg, Germany; paul.schnitzler@med.uni-heidelberg.de; 5 Clinic for Gastroenterology and Hepatology, University Hospital Essen, 45147 Essen, Germany; wedemeyer.heiner@mh-hannover.de; 6 Laboratoire de Microbiologie Clinique, Hôpital Avicenne, APHP, 93000 Bobigny, France; frederic.legal@aphp.fr (F.L.G.); emmanuel.gordien@aphp.fr (E.G.); 7 Department of Gastroenterology, University of Ankara, 06560 Ankara, Turkey; cihan.yurdaydin@medicine.ankara.edu.tr; 8 Department of Gastroenterology and Hepatology, Koç University Medical School, 34450 Istanbul, Turkey

**Keywords:** hepatitis delta virus, recombinant HDAg, pan-genotypic detection, anti-HDV, diagnostics, rapid test, lateral flow assay, point-of-care

## Abstract

Hepatitis Delta virus (HDV) is a satellite of the Hepatitis B virus (HBV) and causes severe liver disease. The estimated prevalence of 15–20 million infected people worldwide may be underestimated as international diagnostic guidelines are not routinely followed. Possible reasons for this include the limited awareness among healthcare providers, the requirement for costly equipment and specialized training, and a lack of access to reliable tests in regions with poor medical infrastructure. In this study, we developed an HDV rapid test for the detection of antibodies against the hepatitis delta antigen (anti-HDV) in serum and plasma. The test is based on a novel recombinant large hepatitis delta antigen that can detect anti-HDV in a concentration-dependent manner with pan-genotypic activity across all known HDV genotypes. We evaluated the performance of this test on a cohort of 474 patient samples and found that it has a sensitivity of 94.6% (314/332) and a specificity of 100% (142/142) when compared to a diagnostic gold-standard ELISA. It also works robustly for a broad range of anti-HDV titers. We anticipate this novel HDV rapid test to be an important tool for epidemiological studies and clinical diagnostics, especially in regions that currently lack access to reliable HDV testing.

## 1. Introduction

Hepatitis delta virus (HDV) infections present the most severe health burden among viral liver diseases. As a satellite virus of the Hepatitis B virus (HBV), transmission takes place via co-infection with HBV or super-infection of patients with chronic hepatitis B (CHB). Persisting infection leads to the establishment of chronic hepatitis D (CHD), which significantly increases the risk of cirrhosis, hepatocellular carcinoma, and acute liver failure, leading to a reduced life expectancy [1,2,3,4]. HDV is a circular, single-stranded, negative-sense RNA virus composed of 1679 nucleotides that codes for two isoforms of a single protein called the hepatitis delta antigen (large: L-HDAg, small: S-HDAg) [5,6,7,8,9]. For viral entry into hepatocytes, HDV requires the integration of the Hepatitis B virus S-antigen (HBsAg) into its envelope [10,11,12]. Antibodies against HDAg (anti-HDV), including IgM and IgG, are not neutralizing but arise in most infected patients [13,14]. Up until recently, HDV therapy was limited to treatment with pegylated interferon-alpha, a regimen with some efficacy but a rare long-term virological response [15,16]. Vaccination against HBV also protects against HDV co-infection, but not against HDV super-infection of HBV carriers. Furthermore, vaccination coverage gaps remain worldwide [17].

The symptoms for HDV co- or super-infection are comparable to HBV mono-infection and can easily be missed. Therefore, international guidelines recommend follow-up testing for HDV in HBsAg-positive individuals with CHB during their first assessment [18,19]. The direct detection of HDV in blood is performed via qRT-PCR, but the genetic diversity of HDV can lead to false negatives [20]. Recently, a consensus commercial kit has been described [21]. As PCR testing only detects patients with active HDV RNA replication, which can be suppressed during therapy, more robust and cost-efficient serological tests for anti-HDV are used for screening purposes [22]. Worldwide, 250 million people are estimated to be HBsAg-positive and are eligible for HDV diagnostics [17]. However, several studies suggest that only a fraction of these patients are tested for anti-HDV, such that HDV infection remains largely underdiagnosed. Retrospective analyses in developed countries revealed low clinical testing rates of 8% in the US [23], 35% in Greece [24], 40% in the UK [25], and 47% in Germany [26]. In many developing countries, HBsAg-patients are not tested for HDV at all [27,28]. Reasons for these trends are diverse and may vary across countries with different medical systems and infrastructures. In general, the awareness of HDV is poor compared to other hepatitis viruses, such as HBV or HCV. This applies to both developed and developing countries and may partly be related to the lack of curative therapy in the past. As several promising antiviral drugs are currently being evaluated in advanced phases of clinical trials [29,30,31], or were recently approved (Bulevirtide), this may soon change. Insufficient testing rates may also be linked to the extensive requirements of the current mode of testing. Conventional anti-HDV detection assays are ELISA-, RIA-, or CLIA-based. These tests require time-consuming experimental protocols performed in a specialized laboratory environment with expensive equipment and trained medical staff [32]. As these needs cannot be met in countries with poor medical infrastructure, the establishment of routine HDV testing is hampered.

The recent developments of antiviral HDV therapies have increased the priority to rapidly identify HDV-infected individuals and to provide access to HDV diagnostics in countries with poor medical infrastructure. Therefore, we developed a point-of-care (POC) device for the rapid and reliable detection of anti-HDV in serum or plasma (HDV rapid test). The test is based on a lateral flow assay (LFA) using a novel, recombinant, and pan-genotypic L-HDAg. The device is easy-to-use, does not require laboratory equipment, and can be applied with minimal training. Therefore, it can be used in a de-centralized manner in both clinical settings and epidemiological studies.

## 2. Materials and Methods

### 2.1. Preparation of Recombinant, Pan-Genotypic HDAg

The recombinant, pan-genotypic L-HDAg (rL-HDAg) was derived from the consensus sequence of 54 published and unpublished HDAg sequences comprising all eight genotypes. In total, seventeen genotype 1, three genotype 2, two genotype 3, two genotype 4, four genotype 5, three genotype 6, two genotype 7, three genotype 8, and eighteen sequences of undefined genotype were aligned. The resulting sequence was manually adjusted for non-consensus amino acids, optimized for bacterial expression, His-tagged, and integrated into a pET construct containing an Ampicillin resistance gene (25 kDa, pI = 9.9). rL-HDAg was expressed in pLys bacteria upon induction with 0.7 mM IPTG and incubated for 3 h at 37 °C. The cells were then pelleted, washed, and resuspended in PBS supplemented with 1:200 Halt^TM^ protease inhibitor (Thermo Fisher Scientific, Waltham, MA, USA). Next, the cells were lysed, ultracentrifuged, and the pellets were resuspended in lysis buffer (8 M urea, 50 mM Tris, 200 mM NaCl, 30 mM imidazole, 1:200 Halt^TM^ protease inhibitor, 5 mM DTT, pH 7.4). The resuspension was pushed through 1.1 mm and 0.8 mm needles, ultracentrifuged, and the supernatant was filtered through a 0.45 µm filter membrane. rL-HDAg was further purified on the Äkta Pure 25 (GE Healthcare, Chicago, IL, USA) using a HisTrap^TM^ HP column (GE Healthcare, Chicago, IL, USA) with urea-containing binding buffer (5 M urea, 50 mM Tris, 200 mM NaCl, 30 mM imidazole, pH 7.4) and elution buffer (5 M urea, 50 mM Tris, 200 mM NaCl, 500 mM imidazole, pH 7.4). Fractions with high protein content were pooled and concentrated to the desired volume. The protein concentration was determined via SDS-PAGE and Coomassie R-250 staining using a BSA standard. Purified rL-HDAg was stored in elution buffer for further experiments.

### 2.2. Preparation of HDV Rapid Test

A sample pad, conjugate pad (Ahlstrom, Helsinki, Finland), membrane (GE Healthcare, Chicago, IL, USA), and absorbent pad (Sigma-Aldrich, Darmstadt, Germany) were cut into pieces of 1.8 × 10 cm, 0.8 × 10 cm, 2.5 × 10 cm, and 1.7 × 10 cm, respectively. Conjugate pads were pre-treated with sample pad buffer (0.5% *w*/*v* casein sodium salt, 0.2% *v*/*v* Tween^®^ 20, 0.1% *w*/*v* sodium azide, PBS) and dried for 1.5–2 h at 40 °C with low humidity. 1:2 diluted gold-conjugated goat anti-human IgG OD15 (BioAssay Works, Ijamsville, MD, USA) in conjugate pad buffer (20% *w*/*v* D(+)-saccharose, 5% *w*/*v* trehalose dihydrate, 0.1% *w*/*v* sodium azide, PBS) was added, using a total volume of 280 µL. A total of 10 µL of 2 mg/mL rL-HDAg in dilution buffer (5 M urea, 50 mM Tris pH 9.0, 200 mM NaCl, 20 mM imidazole) and 1.5 mg/mL donkey anti-goat IgG (H+L)/7S (Dianova, Hamburg, Germany) in PBS were spotted on the membrane using an HPTLC applicator (biostep^®^, Burkhardtsdorf, Germany) at a spotting rate of 1 µL per cm. For the multiplexed LFA detecting both HBsAg and anti-HDV, an additional test line was spotted with 2 mg/mL mouse anti-HBsAg mAb (Fitzgerald, North Acton, MA, USA). The conjugate pad for the multiplex test was soaked with a mixture of gold-conjugated mouse anti-HBsAg (Fitzgerald, North Acton, MA, USA) and gold-conjugated goat anti-human IgG OD15 (BioAssay Works, Ijamsville, MD, USA). Blocking was performed with 2% BSA in PBST for 15 min at room temperature and 15 min at 40 °C. Sample pads were treated with sample pad buffer. Membranes, sample pads, and conjugate pads were dried for 3 h at 40 °C with low humidity. For test strip assembly, three 10 cm sample pads, conjugate pads, membranes, and absorbent pads were glued on a 30 cm backing card (DCN Diagnostics, Carlsbad, CA, USA) and 4 mm test strips were cut using an adjustable paper cutter. The test strips were assembled into plastic cassettes (Shanghai Jieyi Biotech, Shanghai, China) and stored with desiccant in UV-protected air-tight plastic bags at 4 °C.

### 2.3. Detection of Anti-HDV with the HDV Rapid Test

For the detection of anti-HDV in serum or plasma using the novel HDV rapid test, samples were diluted 1:8 in running buffer (0.3% *w*/*v* casein sodium salt, 5% *w*/*v* trehalose dihydrate, 0.1% *w*/*v* sodium azide, PBS) in a total volume of 80 µL. Diluted samples were applied to the sample pad of the test strips and results were recorded by photo after 20 min.

### 2.4. Detection of Anti-HDV by Semi-Quantitative ELISA

ELISA plates were coated with 50 µL of 1 µg/mL rL-HDAg in coating buffer (13 mM Na_2_CO_3_, 88 mM NaHCO_3_, pH 9.2) and blocked with 200 µL/well blocking buffer (1% *w*/*v* casein sodium salt, 0.05% *v*/*v* Tween^®^ 20, PBS). Patient sera were serially diluted 1:8 in dilution buffer (0.1% *w*/*v* casein sodium salt, 0.05% *v*/*v* Tween^®^ 20, PBS), starting at a 1:100 dilution. A total of 50 µL/well was incubated for 1 h at 37 °C. All washing steps were performed with 200 µL/well washing buffer (0.05% Tween^®^ 20 *v*/*v*, PBS). Secondary binding was performed with 1:10,000 HRP-conjugated goat anti-human IgA/IgG/IgM (Jackson Immunoresearch, West Grove, PA, USA) in dilution buffer for 1 h at 37 °C. Signals were obtained by addition of 100 µL/well TMB substrate (Thermo Fisher Scientific, Waltham, MA, USA) for 10 min and reactions were stopped with 100 µL/well 1 M H_3_PO_4_. The OD450 was quantified using the EnVision HTS multilabel reader (Perkin Elmer, Waltham, MA, USA).

### 2.5. Study Population

The HDV rapid test was validated on 474 patient samples, comprising 332 anti-HDV-positive and 142 anti-HDV-negative sera or plasmas against all HDV genotypes except genotype 4. The samples were collected from clinical and commercial vendors, including the University Hospital Heidelberg and the Heidelberg blood bank (Heidelberg, Germany), the Hanover Medical University (Hanover, Germany), the University of Ankara Medical School (Ankara, Turkey), and BIOMEX (Heidelberg, Germany). All HBsAg-positive samples were previously tested for anti-HDV in the respective facilities using diagnostic gold-standard ELISAs (ETI-AB-DELTAK-2 anti-HDV test kit, DiaSorin, Saluggia, Italy or HDV Ab assay, Diapro Diagnostic Bioprobes, Sesto San Giovanni, Italy). For 154 samples, the RNA status was known.

### 2.6. Statistics

The performance of the HDV rapid test was measured as its sensitivity and specificity compared to a current diagnostic gold-standard ELISA. The Wilson score interval was used to calculate 95% confidence intervals (CI).

## 3. Results

### 3.1. Development of a Recombinant, Pan-Genotypic HDAg

**Figure 1:**
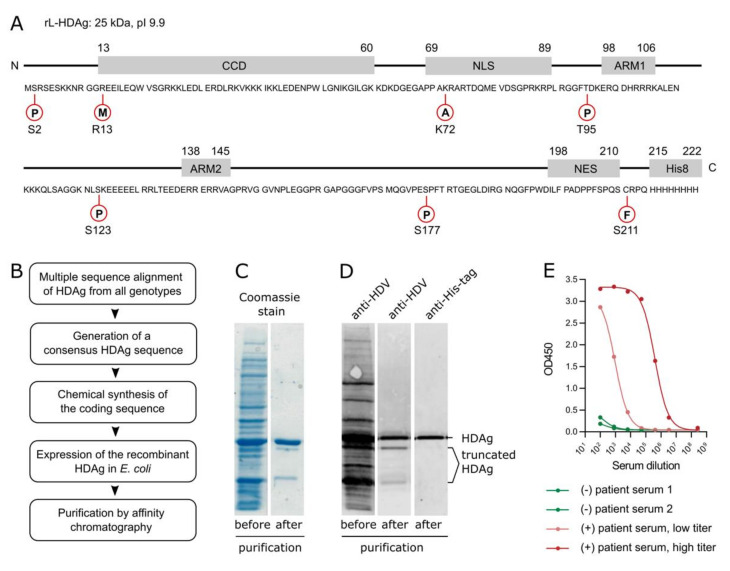
Development of a pan-genotypic recombinant rL-HDAg. (**A**) Optimized consensus sequence of rL-HDAg with a His-tag. All critical features and residues for post-translational modification were maintained. (**B**) Schematic of the development of rL-HDAg. The consensus sequence was derived from a multiple sequence alignment comprising 54 HDAg sequences of all HDV genotypes. (**C**) Purification of rL-HDAg and Coomassie staining. rL-HDAg was purified from bacterial pellets under denaturing conditions using a HisTrap. (**D**) Detection of rL-HDAg via Western blotting. rL-HDAg was detected by the anti-HDV-positive patient serum VUDA. (**E**) Antigenicity of rL-HDAg in a semi-quantitative in-house ELISA using anti-HDV-positive (high titer, low titer) and anti-HDV-negative patient sera.

In this study, we show the development of an HDV rapid test. The test is based on a lateral flow assay which uses a novel, pan-genotypic rL-HDAg for the detection of anti-HDV in serum or plasma. The rL-HDAg was derived from a consensus sequence of 54 published and non-published HDAg sequences to achieve pan-genotypic activity. It codes for a 25 kDa His-tagged protein with an isoelectric point of 9.9. All known functional elements and post-translational modification sites of the viral HDAg were maintained (Figure 1A). The chemically synthesized sequence was expressed in *E. coli*, and the rL-HDAg was purified via affinity chromatography under denaturing conditions (Figure 1B). Using Coomassie staining and Western blotting, we confirmed that the rL-HDAg protein is highly pure and does not contain any protein contaminations, besides two C-terminally truncated isoforms of rL-HDAg that have lost the His-tag (Figure 1C,D). To test the antigenicity of the novel rL-HDAg, we performed a semi-quantitative ELISA using anti-HDV-negative and -positive patient sera at high and low titers (Figure 1E). In a proof-of-principle experiment, we show that rL-HDAg binds to serum anti-HDV in a concentration-dependent manner and does not cross-react with anti-HDV-negative patient serum. Therefore, we conclude that the novel rL-HDAg is suitable for detecting anti-HDV in serum or plasma samples.

### 3.2. Development of the HDV Rapid Test and Proof-of-Principle

**Figure 2:**
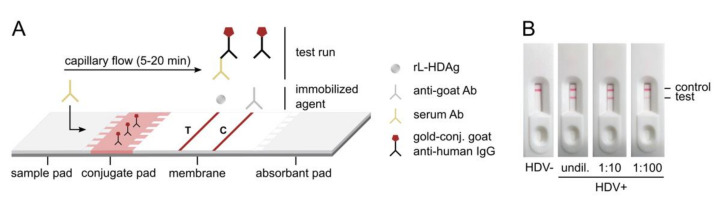
Principle of the HDV rapid test. (**A**) Schematic of the lateral flow assay for the detection of anti-HDV in patient serum. (**B**) Proof-of-principle of the HDV rapid test using anti-HDV-positive and -negative patient sera.

The novel rL-HDAg was incorporated in a lateral flow assay to create a rapid point-of-care test for the pan-genotypic detection of anti-HDV (Figure 2A). In this assay, rL-HDAg is spotted as a test line on the membrane and binds to anti-HDV in the tested patient sample. As anti-HDV is previously labeled with gold-conjugated goat anti-human IgG present in the conjugate pad, a red test line appears for visual detection. On the control line, immobilized anti-goat binds to the gold-conjugates in a sample-independent manner, serving as an internal control for the test. The flow of the patient sample through the test strip is ensured by capillary forces and takes 5–20 min. For proof-of-principle experiments, the rapid test strips were assembled into plastic cassettes and anti-HDV-positive and -negative patient sera were applied (Figure 2B). We show that the novel HDV rapid test can effectively detect anti-HDV in patient serum and does not cross-react with negative serum. Strikingly, test positivity was maintained for a 1:10 and 1:100 dilution of the positive serum, indicating that the test is highly sensitive. To evaluate this in more detail, we complemented anti-HDV-negative patient serum with humanized mouse anti-HDAg mAb. We found that the minimum concentration resulting in a positive HDV rapid test result was 1.5 µg/mL, equivalent to 15 ng monoclonal antibody per test (data not shown).

### 3.3. Test Validation

**Figure 3:**
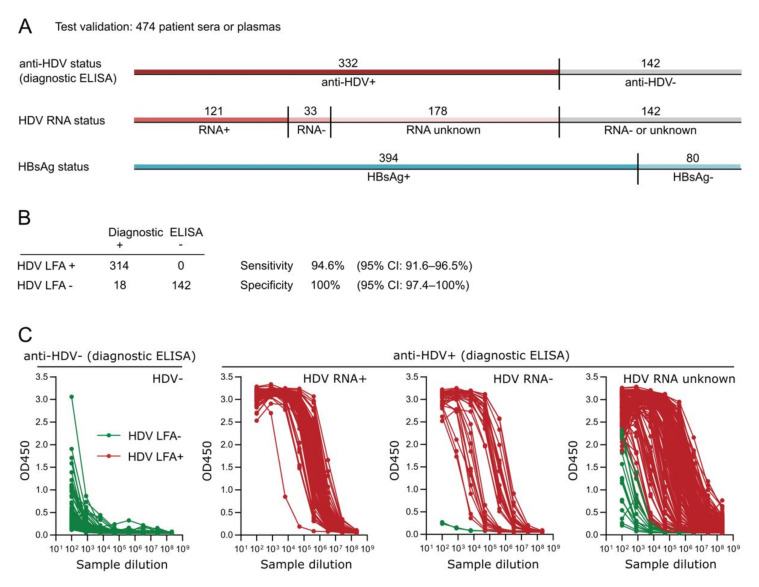
Test validation of the HDV rapid test. (**A**) Characterization of test validation sera or plasmas according to their pre-known anti-HDV status (ETI-AB-DELTAK-2 anti-HDV ELISA, DiaSorin, Saluggia, Italy or HDV Ab assay, Diapro Diagnostic Bioprobes, Sesto San Giovanni, Italy), HDV RNA status, and HBsAg status. Patient sera were collected from clinical and commercial vendors, including the University Hospital Heidelberg and the Heidelberg blood bank (Heidelberg, Germany), the Hanover Medical University (Hanover, Germany), the University of Ankara Medical School (Ankara, Turkey), and BIOMEX (Heidelberg, Germany). (**B**) Sensitivity and specificity of the HDV rapid test compared to the gold-standard ELISA. (**C**) Semi-quantification of anti-HDV in all 474 patient sera using an in-house ELISA. All sera were previously tested positive or negative for anti-HDV using a commercial gold-standard ELISA. The same samples were tested for anti-HDV using the HDV rapid test. Qualitative HDV rapid test results (positive: green; negative: red) are overlaid with the semi-quantitative in-house ELISA results.

To determine the relative performance of the rapid test compared to a gold-standard ELISA assay, we conducted a validation study using a cohort of 474 pre-characterized patient sera or plasmas (Figure 3A). The samples were previously tested positive (332 samples) or negative (142 samples) for anti-HDV using a commercially available diagnostic ELISA. We re-tested all samples using our novel HDV rapid test (Figure 3B). Out of 332 anti-HDV-positive patient samples in the diagnostic ELISA, 314 samples were also positive in the HDV rapid test, while 18 samples were negative. This corresponds to a sensitivity of 94.6% (95% CI: 91.6–96.5%). Furthermore, we found the specificity to be 100% (95% CI: 97.4–100%), as all 142 anti-HDV-negative samples in the diagnostic ELISA were also negative in the HDV rapid test. No cross-reaction with HBsAg was observed.

To understand the performance of the HDV rapid test in more detail, we semi-quantified the anti-HDV titers in all patient samples using an in-house ELISA and compared them to the results of the diagnostic ELISA and the HDV rapid test (Figure 3C). Interestingly, all samples that tested negative in the HDV rapid test but positive in the diagnostic ELISA had very low anti-HDV titers that were comparable to the unspecific binding signals in the 142 negative controls. Furthermore, all ambiguous samples were never confirmed to be HDV RNA-positive but had either an unknown HDV RNA status (17 samples) or were HDV RNA-negative (one sample). We also saw that HDV RNA-positive samples generally displayed high titers of anti-HDV, while the HDV RNA-negative samples were subdivided into three cohorts with either high, medium, or low anti-HDV titers. Our results indicate that the HDV rapid test can successfully identify positive samples with a broad range of anti-HDV levels but does not react to samples with low ELISA signals in the negative control range. It currently remains unclear whether these samples are true positives that are missed by our HDV rapid test or false positives of the diagnostic ELISA.

### 3.4. Pan-Genotypic Activity of the HDV Rapid Test and Multiplexing with HBsAg Detection

**Figure 4:**
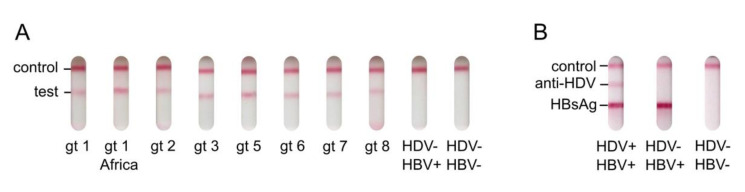
Pan-genotypic activity of the HDV rapid test and HBsAg multiplexing. (**A**) Pan-genotypic activity of the HDV rapid test. Anti-HDV-positive samples of patients that were infected with specific HDV genotypes were run on the HDV rapid test. Anti-HDV-negative samples of HBsAg-positive and -negative patients were used as controls. (**B**) Multiplexing of the HDV rapid test with the detection of HBsAg. Anti-HBsAg was spotted as a third line on the HDV rapid test for the detection of HBsAg in patient serum or plasma. The conjugate pad was treated with a mixture of gold-conjugated goat anti-human IgG and gold-conjugated mouse anti-HBsAg. The result of a single proof-of-principle experiment is shown.

The rL-HDAg used in the HDV rapid test was designed to detect anti-HDV against all HDV genotypes. To test the pan-genotypic activity, we collected sera or plasmas from patients with known HDV genotype 1, 2, 3, 5, 6, 7, or 8 infections. By re-testing these samples for anti-HDV using the HDV rapid test, we confirmed that the test is reactive to all HDV genotypes (Figure 4A). Unfortunately, a genotype 4 sample was not available for testing.

We already showed that anti-HDV can be successfully detected in the lateral flow assay format of our HDV rapid test. As HDV can only occur together with HBV, we were interested in testing whether the detection of HBsAg and anti-HDV can be multiplexed in our assay. Therefore, we added a second HBsAg test line to the test strip containing immobilized mouse anti-HBsAg mAb. Strikingly, this set-up was able to independently detect anti-HDV and HBsAg in pre-characterized patient samples (Figure 4B). This proof-of-principle experiment shows that the HDV rapid test can easily be multiplexed with the detection of HBsAg to generate a one-step test to distinguish HBV mono- from HBV-HDV co-infection.

## 4. Discussion

The rapid and reliable diagnosis of HDV infections is of high priority to make patients eligible for therapy. However, international HDV testing guidelines are insufficiently followed, possibly due to a lack of awareness and costly, time-consuming diagnostic procedures requiring specialized equipment and training. Here, we developed a rapid HDV test for the detection of anti-HDV in serum or plasma within 20 min. The test is based on an easy-to-use and cost-effective lateral flow assay that can be applied in decentralized settings and regions with poor medical infrastructure [26]. Therefore, it can broaden the access to reliable HDV diagnostics worldwide and ease the burden of this life-threatening disease. The clinical relevance of LFA-based antibody tests was highlighted during the SARS-CoV-2 pandemic, when conventional routine lab testing was overloaded and not available in multiple settings and regions.

The HDV rapid test incorporates a novel pan-genotypic rL-HDAg that can bind anti-HDV against all HDV genotypes (genotype 4 not tested) in a highly specific and concentration-dependent manner. This is of especially high relevance in regions like Africa, Asia, and South America, where strains of almost all HDV genotypes are spreading. Interestingly, antigen-antibody binding on the rapid test was possible despite using denatured rL-HDAg. This is in agreement with previous studies which found that large segments of the HDAg protein sequence are intrinsically disordered but maintain the ability to form protein-protein interactions [33,34,35,36].

The sensitivity of antibody rapid tests can be lower compared to ELISA-based assays due to the visual detection of colloidal gold conjugates forming the test line. On the other hand, ELISAs are more prone to lower specificity due to unspecific binding signals. In this study, we provide a comprehensive performance evaluation of the HDV rapid test compared to a diagnostic ELISA using a cohort of 474 pre-characterized samples. As expected, we found that the LFA has a higher specificity (100% vs. 99.0%) but a lower sensitivity (94.6% vs. 99.4%) compared to the diagnostic ELISA used. However, when evaluating the samples with non-corresponding test results in more detail, we found that they had very low anti-HDV ELISA signals closely resembling the included negative controls. Furthermore, these samples had not tested positive for HDV RNA to confirm HDV infection. Therefore, it currently remains unclear whether the observed differences were due to false negative test results of the HDV rapid test, or whether these samples were wrongfully considered positive by the diagnostic ELISA. To increase the objectivity and readability of LFA tests, their read-out can be automated by digital instruments. These hand-held or benchtop devices measure the colorimetric change on the test and control lines and generate a semi-quantitative test result. Such technology can assist medical professionals in their daily diagnostic routine, or operators of epidemiological studies.

Low HDV testing rates may have affected our understanding of the global prevalence and distribution of HDV. The World Health Organization (WHO) estimates that 5% of all HBsAg-positive people are infected with HDV, resulting in a total number of 15–20 million HDV infections worldwide. HDV hotspots were recorded in Mongolia, the Amazon basin, and the Mediterranean basin, as well as in some countries in Africa and western Asia [27,28]. However, recent meta-analyses highlighted that for many countries the HDV prevalence remains entirely unknown, is insufficiently mapped, or only represents specific regions or risk-groups. While one meta-analysis study concluded that the HDV prevalence may be highly underestimated [27], another study supported the WHO numbers [28]. Hence, the true HDV prevalence remains to be elucidated and may depend significantly on future epidemiological studies in countries that have previously lacked access to HDV diagnostics.

## 5. Conclusions

The novel HDV rapid test provides us with an important tool for epidemiological HDV research, especially in countries with poor medical infrastructure. It may also fill a critical gap in clinical HDV diagnostics in the future.

## 6. Patents

The *E. coli* derived recombinant L-HDAg with a non-naturally occurring consensus sequence of all eight HDV genotypes is protected by the patent application WO2019219840A1.

## Data Availability

The data presented in this study are available on request from the cor- responding author. The data are not publicly available due to data privacy and ethical considerations.
